# On the daily cycle of mesoscale cloud organization in the winter trades

**DOI:** 10.1002/qj.4103

**Published:** 2021-06-09

**Authors:** Jessica Vial, Raphaela Vogel, Hauke Schulz

**Affiliations:** ^1^ LMD/IPSL Sorbonne Université, CNRS Paris France; ^2^ Max Planck Institute for Meteorology Hamburg Germany

**Keywords:** daily cycle, mesoscale organization, observations, trade‐wind cumuli

## Abstract

How spatial organization of clouds at the mesoscale contributes to the daily cycle of shallow cumulus clouds and precipitation is here explored, for the first time, using three years of high‐frequency satellite‐ and ground‐based observations. We focus on the four prominent patterns of cloud organization – Sugar, Gravel, Flowers and Fish – which were found recently to characterize well the variability of the North Atlantic winter trades. Our analysis is based on a simple framework to disentangle the parts of the daily cycle of trade‐wind cloudiness that are due to changes in (a) the occurrence frequency of patterns, and (b) cloud cover for a given pattern. Our investigation reveals that the contribution of mesoscale organization to the daily cycle in cloudiness is largely mediated by the frequency of pattern occurrence. All forms of mesoscale organization exhibit a pronounced daily cycle in their frequency of occurrence, with distinct 24‐hr phasing. The patterns Fish and Sugar can be viewed as *daytime* patterns, with a frequency peak around noon for Fish and towards sunset for Sugar. The patterns Gravel and Flowers appear instead as *night‐time* patterns, with a peak occurrence around midnight for Gravel and before sunrise for Flowers. The cloud cover for a given pattern, however, always maximizes at night‐time (between 0000 and 0300 hr), regardless of the specific pattern. Analyses of the role of large‐scale environmental conditions shows that the near‐surface wind speed can explain a large part of the diurnal variability in pattern frequency and cloudiness.

## Graphical abstract

How spatial organization of clouds at the mesoscale contributes to the daily cycle of shallow cumulus clouds and precipitation is explored, for the first time, using three years of high‐frequency satellite‐ and ground‐based observations. The focus is on the four prominent patterns of cloud organization – Sugar, Gravel, Flowers and Fish – which were found recently to characterize well the variability of the North Atlantic winter trades. The figure shows that all forms of mesoscale organization exhibit a pronounced daily cycle in their frequency of occurrence, with distinct 24‐hr phasing. The pattern Fish and Sugar can be viewed as daytime patterns, while Gravel and Flowers appear instead as night‐time patterns.

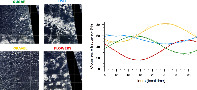

## 1 INTRODUCTION

As one of the most fundamental modes of tropical climate variability, the daily cycle has been thoroughly studied for many cloud types except, surprisingly, for shallow cumuli in the trade‐wind regime. Indeed, the daily cycle of trade‐wind cumuli has only been recently described in some detail by Vial *et al*., (2019), 40 years after it was first documented (Nitta and Esbensen, 1974; Brill and Albrecht, 1982). In typical conditions of the North Atlantic winter trades, cloudiness overall maximizes at the end of the night and is minimum in the afternoon. This daily cycle reflects the evolution of two distinct cloud populations: (a) a daytime population of non‐precipitating small cumuli, which peaks around sunset and has weak vertical extents of only a few hundred metres above the Lifting Condensation Level (LCL), and (b) a night‐time population of deeper precipitating clouds, peaking just before sunrise, which is often accompanied by a stratiform cloud shield spreading below the trade inversion (Vial *et al*., 2019). In storm‐resolving and large‐eddy simulations run over large domains, these two cloud populations seem to be associated with distinct spatial organizations at the mesoscale (figure 4 in Vial *et al*., 2019). The daytime population exhibits a multitude of small cumuli scattered in a regular pattern over hundreds (or even thousands) of kilometres, while the night‐time field tends to show fewer and larger cloud clusters, sometimes organized along extensive lines or arcs reminiscent of gust fronts accompanying rainfall‐generated cold pools (Zuidema *et al*., 2012; Ruppert and Johnson, 2016). However, whether this visual impression from the simulations is realistic and systematic, and whether this type of spatial organization plays a role in the daily cycle of trade‐wind clouds and convection, remain open questions.

**FIGURE 1:**
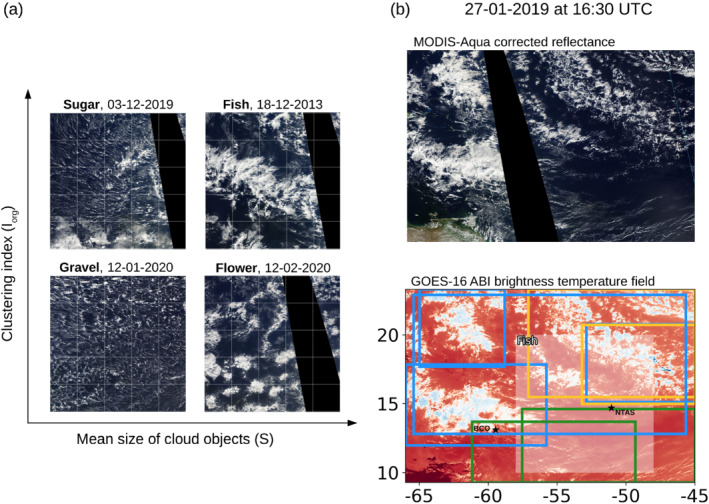
(a) MODIS‐Aqua satellite images from NASA Worldview, illustrating the four prominent mesoscale cloud organization patterns of the North Atlantic trade‐wind region over the 10${\;}^{\circ }\times 10^{\circ }$ $I_{\text{org}}/S$ classification domain. The images are positioned in the four quadrants defined by the lower and upper terciles of the S and $I_{\text{org}}$distributions. (b, lower panel) Illustration of NN‐detected pattern rectangles using the GOES‐16 ABI $T_{b}$ field over the 14${\;}^{\circ }\times 21^{\circ }$ classification domain (Gravel in yellow, Fish in blue and Sugar in green) and the corresponding $I_{\text{org}}/S$ classification (Fish) over the smaller domain (in grey shading). (b, upper panel) is the corresponding visible MODIS image from NASA Worldview. The locations of the two sites, BCO and NTAS, are shown in (c). Note that the NN classification domain is positioned such that BCO and NTAS are equally distant from the lateral edges of the domain, and the $I_{\text{org}}/S$ domain is chosen as in Bony *et al.*, (2020), upwind of Barbados

It has long been recognized that shallow convection can organize spatially into various patterns, the most classical forms being cloud streets, closed or open cells in cold air outbreaks or in subtropical upwelling areas (Atkinson and Wu, 1996; Wood and Hartmann, 2006; McCoy *et al*., 2017). In the North Atlantic trades, different environmental conditions (e.g., warmer sea surface, weaker subsidence) give rise to other forms of cloud organization, which have been recently discovered and characterized in satellite‐ and ground‐based observations (Stevens *et al*., 2020; Bony *et al*., 2020; Schulz *et al*., 2021). Each organization pattern features a specific type of cloud and a specific spatial layout of the cloud field on scales from 20 to 2,000 km. The patterns range from isolated, shallow, non‐precipitating cumulus clouds (*Sugar* organization), to precipitating cumuli forming along lines or arcs defining gust fronts (*Gravel* organization), and to organized structures of deeper precipitating cumuli with a stratiform cloud layer at their top that can extend up to hundreds of kilometres and that are separated by large and well‐defined cloud‐free areas (*Fish* and *Flowers* organizations). These four patterns are illustrated in Figure 1.

The fact that the daily cycle and mesoscale organization of trade‐wind cumuli have only recently been discovered (or revived) clearly shows that there is a significant gap in our understanding of the dynamics of trade‐wind shallow convection and clouds. This is of particular concern as the coupling between marine shallow trade‐wind clouds and circulation is known to play a central role in the uncertainty of the tropical cloud feedback and climate sensitivity estimated by models (Rieck *et al*., 2012; Brient and Bony, 2013; Sherwood *et al*., 2014; Bony *et al*., 2015; Tomassini *et al*., 2015, Vogel *et al*., 2016, Vial *et al*.,^2016^, ^2017^). To help fill this knowledge gap, and to improve our ability to predict the Earth's climate response to warming, in this article we build upon the work of Vial *et al*., (2019) to investigate more thoroughly the link between the daily cycle and the spatial organization of trade‐wind shallow cumulus convection. To our knowledge, this study is the first to question the role of spatial organization in the daily cycle of shallow cumulus convection. Specifically, we explore whether the observed occurrence of the four aforementioned patterns of organization exhibits variability on the daily time‐scale, and whether the different patterns of organization influence differently the daily cycle of trade‐wind cloudiness and precipitation.

Our analysis combines satellite‐ and ground‐based remote‐sensing observations, as well as insitu surface measurements in the North Atlantic trade‐wind region, windward of Barbados, which is known to be representative of the trade‐wind regime in other ocean basins (Medeiros and Nuijens, 2016; Stevens *et al*., 2016; Rasp *et al*., 2019). We focus on the boreal winter season, when the Intertropical Convergence Zone is at its southernmost position, and thus when shallow cumuli predominate. After a description of the observational datasets and analysis framework (Section 2), we document the daily cycle in the occurrence frequency of mesoscale patterns (Section 3), and investigate the different ways in which the daily cycle of trade‐wind cloudiness and precipitation depend on the mesoscale patterns of organization (Section 4). Finally, we explore the role of the large‐scale environment in the variability of pattern occurrence and cloudiness at the daily time‐scale (Section 5). Our conclusions are presented in Section 6.

## 2 OBSERVATIONAL DATASETS AND METHODOLOGY

We use satellite‐ and ground‐based remote‐sensing and insitu observations over the tropical Atlantic Ocean around Barbados during the boreal winter months (DJFM) from 1 January 2018 to 31 March 2020. The different datasets and their use are described in the following subsections.

### 2.1 GOES‐16 satellite data

The Geostationary Operational Environmental Satellite (GOES)‐16 is the current satellite in the GOES‐East location (centred at 75.2°W), providing data since December 2017. We use 30 min infrared (13 μm) brightness temperature ($T_{b}$) at a spatial resolution of 2 km from the Advanced Baseline Imager (ABI) Level 1b data product (GOES‐R Calibration Working Group, 2017).

#### 2.1.1 Cloud organization classification

Two different approaches are employed here to classify mesoscale patterns of shallow cloud organization using GOES‐16 ABI data.

**FIGURE A1:**
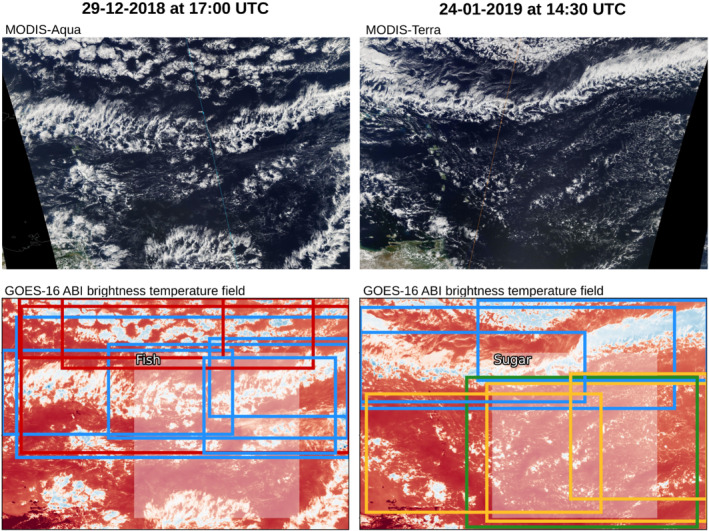
As Figure 1b, but for different dates. The colour code for the rectangles is the same as the one used throughout the manuscript: red is for Flowers, blue for Fish, yellow for Gravel, green for Sugar. The $I_{\text{org}}/S$ classification is indicated in the grey area

The first method, developed in Bony *et al*., (2020) and referred to here as the $I_{\text{org}}/S$ method, characterizes the organization of a marine shallow cloud population within a fixed 10${\;}^{\circ }\times 10^{\circ }$ domain east of Barbados (48°–58°W, 10°–20°N) based on the mean size (S) and the clustering ($I_{\text{org}}$) of segmented cloud objects, which correspond to pixels for which $280\leq T_{b}\leq 290$ K. The index $I_{\text{org}}$ was defined by Tompkins and Semie (2017) such that $I_{\text{org}}<0.5$ corresponds to a regularly distributed cloud population, $I_{\text{org}}=0.5$ to a random distribution and $I_{\text{org}}>0.5$ to a clustered distribution. The lower and upper terciles of S and $I_{\text{org}}$ distributions are then used to classify the mesoscale patterns into four categories (Figures 1a and A1): Sugar is classified as high $I_{\text{org}}$ and low S; Gravel as low $I_{\text{org}}$ and low S; Fish as high $I_{\text{org}}$ and high S; and Flowers as low $I_{\text{org}}$ and high S. The unclassified pattern (also referred to as the No category in the figures) is defined as all the times when $I_{\text{org}}$ and S fall in the intermediate terciles. Bony *et al*., (2020) provide more details.

The second method, called the NN method, is based on the neural network (NN) algorithm originally developed and trained with visible satellite images in Rasp *et al*., (2019), and adapted to infrared images in Schulz *et al*., (2021). Rectangles of the four cloud organization classes (Sugar, Gravel, Flowers, Fish) are detected over a 14${\;}^{\circ }\times 21^{\circ }$ domain (45°–66°W, 9.3°–23.3°N) including Barbados, and the No category is considered as the remaining part of the domain where none of the four patterns is detected (illustration in Figure 1). Any number of pattern rectangles of various sizes can be detected at a given time (with a minimum rectangle size of about 10% of the domain area), with potential overlaps between them. When overlaps occur between several rectangles of the same pattern (e.g., three overlapping Gravel rectangles), we merge them into one polygon with the pattern area being the union of all overlapping rectangles; thus we only count the overlapping area once. Overlaps can also occur between different labels (e.g., one Fish overlaps with one Gravel rectangle) due to ambiguous forms of organization, or connectivity among patterns (Rasp *et al*., 2019; Stevens *et al*., 2020). This could result either from a weak machine learning prediction (which is affected by the quality of the human labels), or it could have a physical explanation (for instance, the overlaps could occur during transitions between patterns). Figure 1b shows an example of an overlap between Gravel and Fish (on the top‐right corner of the domain), since the Fish here appears as a network of Gravel‐like cold‐pool structures – which can be seen particularly well in the visible image from MODerate‐resolution Imaging Spectroradiometer (MODIS) at the top. In these situations (i.e., of overlaps between different labels), we simply count the total area of all rectangles without removing any overlap. The total area of patterns (including the unclassified pattern) is thus greater than the domain area; we discuss this further in Section 2.1.2.

**FIGURE A2:**
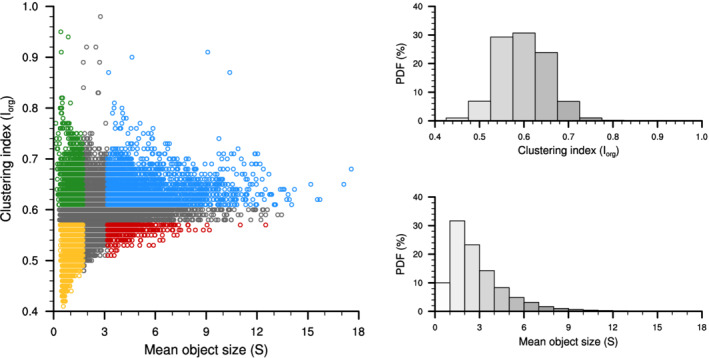
(left) characterization of shallow cloud organization from the thresholding by terciles of the $I_{\text{org}}$ and S distributions. Sugar is shown in green, Gravel in yellow, Flowers in red, Fish in blue, and the undefined regime is shown in grey. (right) distribution functions of the (top) clustering index ($I_{\text{org}}$) and (bottom) mean size of cloud objects (S)

These two classification methods are quite different in nature. $I_{\text{org}}/S$ is based on geometrical and statistical properties of the cloud field, and as such could be considered as the most objective of the two approaches. However, since $I_{\text{org}}$ and S are continuous measures and the patterns can only be robustly identified at the extremes (here, subjectively chosen first and third terciles) of the paired ($I_{\text{org}}$, S) distributions, the inner paired tercile is by default unclassified and marks the regime of transitional or unclear patterns. This transitional regime represents 5/9 of the paired distributions (∼55% of the time), which thus potentially constitutes an important methodological bias in the interpretation of our results (cf. Section 3). The NN method does not suffer from this issue, but given the subjective categories, which human labelers sometimes did not agree on, it can sometimes yield ambiguous classifications as well (cf. Figures 1b and A1 and A2). Stevens *et al*., (2020) and Bony *et al*., (2020) have shown that especially the patterns Fish and Flowers on the one hand, and Gravel and Sugar on the other hand, can be confused. Examples of these two ambiguities are shown in Figure A2.

**FIGURE 2:**
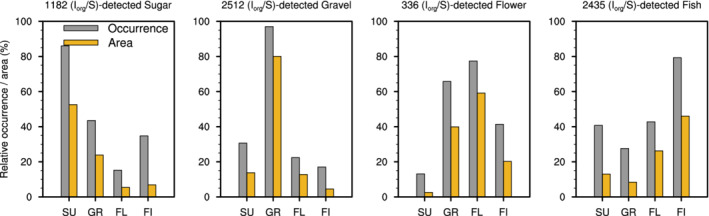
Relative occurrence (grey bars) and area (yellow bars) of NN‐detected patterns overlapping the 10${\;}^{\circ }\times 10^{\circ }$ classification area at times when $I_{\text{org}}/S$ detects a pattern. The frequencies of occurrence are computed with respect to the number of $I_{\text{org}}/S$‐detected patterns (as indicated at the top of each panel), and the areas are computed over the overlapping part of the NN‐detected rectangles with the 10${\;}^{\circ }\times 10^{\circ }$ domain and normalized by the 10${\;}^{\circ }\times 10^{\circ }$ area. The sum of occurrences and areas will thus be greater than 100%

**FIGURE 3:**
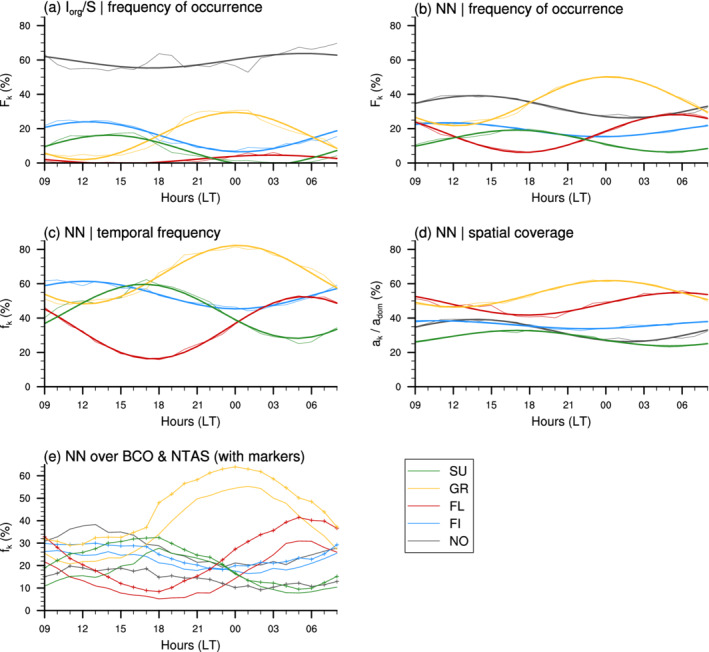
(a, b) daily cycle in the occurrence frequency of mesoscale patterns for Sugar (green), Gravel (yellow), Flowers (red), Fish (blue) and No category (grey), with the first harmonics superimposed in thicker lines. (a) shows the 10${\;}^{\circ }\times 10^{\circ }$ domain‐scale $I_{\text{org}}/S$ pattern frequency and (b) shows the occurrence frequency of NN‐detected patterns within the 14${\;}^{\circ }\times 21^{\circ }$ domain. (c, d) contributions to the NN occurrence frequency owing to (c) the temporal frequency and (d) spatial coverage. (e) temporal frequency of NN‐detected patterns over BCO (solid line) and NTAS (line with markers). Note that, at a single point, the temporal frequency corresponds to the frequency of occurrence

Another difference between NN and $I_{\text{org}}/S$ is that the former can detect several cloud patterns within a domain and therefore does not have to classify a complete fixed domain like $I_{\text{org}}/S$. To compare the two methods, we can ask to which NN‐detected predominant pattern does the domain‐scale pattern identification with $I_{\text{org}}/S$ correspond. To address this question, we compute the relative occurrence and area of NN‐detected patterns overlapping the 10${\;}^{\circ }\times 10^{\circ }$ classification domain at times when $I_{\text{org}}/S$ detects a specific pattern. Figure 2 shows that the two methods result in fairly consistent classifications. That is, when $I_{\text{org}}/S$ detects a specific pattern, about half (or more) of the 10${\;}^{\circ }\times 10^{\circ }$ domain is covered with the NN‐detected patterns of the same category. For instance, in 80% of Sugar cases detected by $I_{\text{org}}/S$, NN detects predominantly Sugar patterns with an average coverage of 50% of the $I_{\text{org}}/S$ domain. In the case of Gravel, nearly all $I_{\text{org}}/S$ classifications correspond to NN‐detected Gravel patterns covering on average 80% of the $I_{\text{org}}/S$ domain. The Flowers pattern is the one for which the correspondence between the $I_{\text{org}}/S$ and NN identifications is the least clear, because of a relatively high occurrence of NN‐detected Gravel (Figure 2, third panel). The exact reason for this ambiguity has not clearly been identified, but it might be related to the thresholding by terciles chosen in Bony *et al*., (2020) and applied here as well. As shown in Figure A1 and A2, the distributions of $I_{\text{org}}$ and S are skewed toward high $I_{\text{org}}$ and low S values, which disfavour the detection of Flowers situations (as also evidenced in Figures 2 and 3a by the relatively small sample size of $I_{\text{org}}/S$‐detected Flowers), and in particular, Flowers situations with large mean cloud size S. This results in the Gravel and Flowers situations to be quite close to each other in the $I_{\text{org}}/S$ space, and thus perhaps less easily distinguishable with this method. Note that the smallest ambiguity in the third panel of Figure 2 occurs for NN‐detected Sugar, which is precisely the furthest from the Flowers pattern in the $I_{org}/S$ space.

The correspondence in pattern detection and classification between the two approaches is thus fairly satisfactory. Nevertheless, as discussed above, each of these two classification methods has its own limitations, and thus both methods will be used in our study in order to more robustly assess the daily cycle of mesoscale cloud organization and its influence on the daily cycle of cloudiness. It is also worth mentioning that the NN approach can offer two additional advantages with respect to $I_{\text{org}}/S$: (a) a more accurate characterization of the atmospheric or surface properties of the patterns (since it does not have to classify an entire domain), and (b) the possibility to select those patterns that are located over instrumented sites to characterize the patterns in greater detail using the ground‐based measurements.

#### 2.1.2 Pattern cloud covers

The shallow cloud cover (CC) is computed from the GOES‐16 ABI $T_{b}$ mask as defined earlier, that is, CC is 1 in pixels for which 280 $\leq T_{b}\leq 290$ K, and 0 otherwise.

When using the $I_{\text{org}}/S$ classification, the cloud cover is averaged over the 10${\;}^{\circ }\times 10^{\circ }$ domain at each timestep and is assigned to one of the four cloud patterns or to the unclassified category. In doing so, the domain‐mean CC averaged over the entire period (DJFM 2018–2020) can be expressed as

$$\begin{array}{ll} \bar{CC}=\underset{k}{\sum }(CC_{k}\times F_{k}) & \end{array}$$

where k refers to pattern labels (SU, GR, FL, FI, NO), $CC_{k}$ is the CC of a given pattern k (also referred to as “pattern‐related cloud cover”) and $F_{k}$ is the frequency of occurrence of pattern k at a given time of the 24‐hr day, such that $\sum_{k}F_{k}=100\%$ across the daily cycle. The product $CC_{k}\times F_{k}$ is the effective contribution of pattern k to the total cloud cover.

The analysis, based on this simple decomposition, allows us to both (a) quantify the relative contribution of mesoscale patterns to the daily cycle in total cloud cover, and (b) disentangle the part of the daily cycle that is due to changes in pattern occurrence frequency and the part due to changes in cloudiness for a given pattern. For reference, we present in Table 1 the daily characteristics of these two main quantities ($F_{k}$ and $CC_{k}$) derived from the different methods, geographical locations and observational datasets (as described below).

**TABLE 1:** Observed daily characteristics of pattern frequency of occurrence ($F_{k}$) and cloud cover ($CC_{k}$), as derived from $I_{\text{org}}/S$ and NN over their respective computational domains and (with NN only) at the location of the intrumented sites BCO and NTAS: diurnal mean (in %), amplitude (amp, in %) and local time of daily maximum (phase, rounded to the nearest hour) – as derived from the first harmonics

	Sugar			Gravel			Flowers			Fish			No		
	mean	amp	phase	mean	amp	phase	mean	amp	phase	mean	amp	phase	mean	amp	phase
**Frequency of occurrence (** $F_{k}$ **)**															
$I_{\text{org}}/S$	7.4	8.8	14	15.7	13.8	00	2.1	2.4	03	15.2	8.8	12	59.5	4.2	05
NN	12.8	6.4	17	36.0	14.3	00	17.2	11.0	06	19.4	4.1	12	32.7	6.5	14
NN‐BCO	16.2	8.4	18	37.1	17.5	00	15.7	12.0	06	21.6	4.6	12	27.3	8.4	13
NN‐NTAS	21.5	11.0	16	46.3	17.6	00	24.2	15.6	05	24.8	5.6	12	14.4	4.3	14
**GOES‐16 ABI cloud cover (** $CC_{k}$ **)**															
$I_{\text{org}}/S$	7.1	1.1	07	13.2	1.2	03	26.9	3.5	02	25.9	5.5	03	19.4	4.0	03
NN	6.5	1.3	01	16.8	2.4	02	26.7	4.3	00	28.2	3.3	00	19.9	3.8	01
NN‐BCO	5.8	1.1	02	15.4	2.5	02	24.8	4.0	02	26.2	3.4	01	—	—	—
NN‐NTAS	6.8	1.8	01	16.6	2.6	02	25.8	4.8	01	27.1	3.7	00	—	—	—
**BCO radar cloud cover (** $CC_{k}$ **)**															
NN‐BCO	20.3	2.3	23	26.3	3.1	23	33.9	6.3	20	36.7	2.7	19	26.7	5.1	01

We follow the same approach when using the NN classification, but because multiple patterns with different sizes can be detected at one timestep over the domain, the frequency of pattern occurrence ($F_{k}$) becomes dependent on both the area of patterns ($a_{k}$) relative to the domain area ($a_{\text{dom}}$) – that is, $a_{k}/a_{\text{dom}}$ – and on their temporal frequency of occurrence ($f_{k}$), such that

$$\begin{array}{ll} F_{k}=f_{k}\times a_{k}/a_{\text{dom}}, & \end{array}$$

$$\begin{array}{ll} \text{where}\;a_{\text{dom}}=\sum_{k}a_{k}\;\text{and thus}\sum_{k}F_{k}=\sum_{k}f_{k}=100\%. & \end{array}$$

**FIGURE A3:**
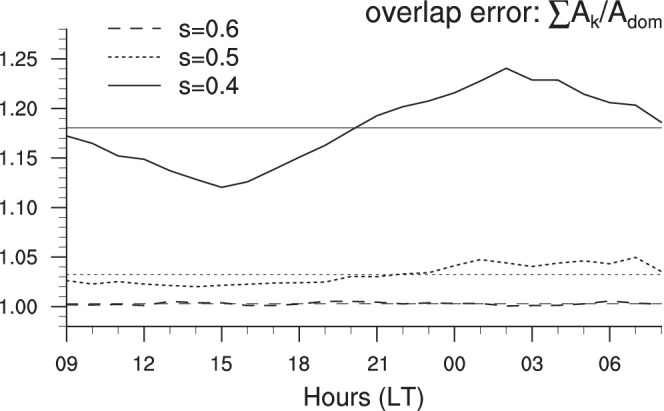
Daily cycle of the *“error”* due to overlaps between several rectangles of different labels for three values of the classification score. The horizontal lines are the daily means of the error. The error is actually the sum of all pattern areas (including the no classification) normalized by the domain area. A value of 1 means no error

**FIGURE A4:**
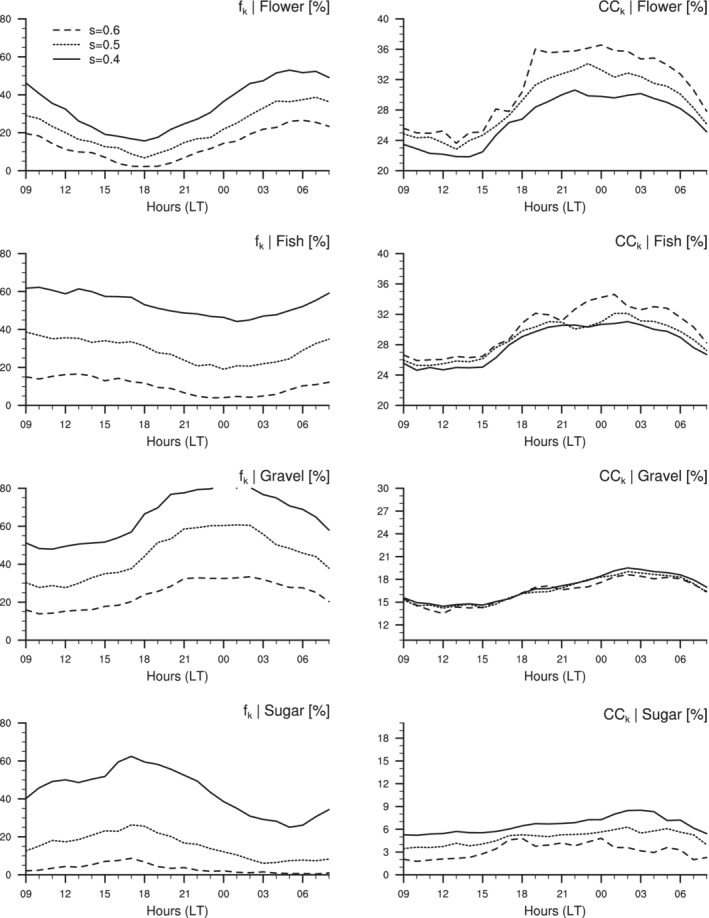
Daily cycle of pattern temporal frequency of occurrence ($f_{k}$, left panels) and pattern cloud cover ($CC_{k}$, right panels) for different values of the agreement score. The default score used in the study is s=0.4

**FIGURE A5:**
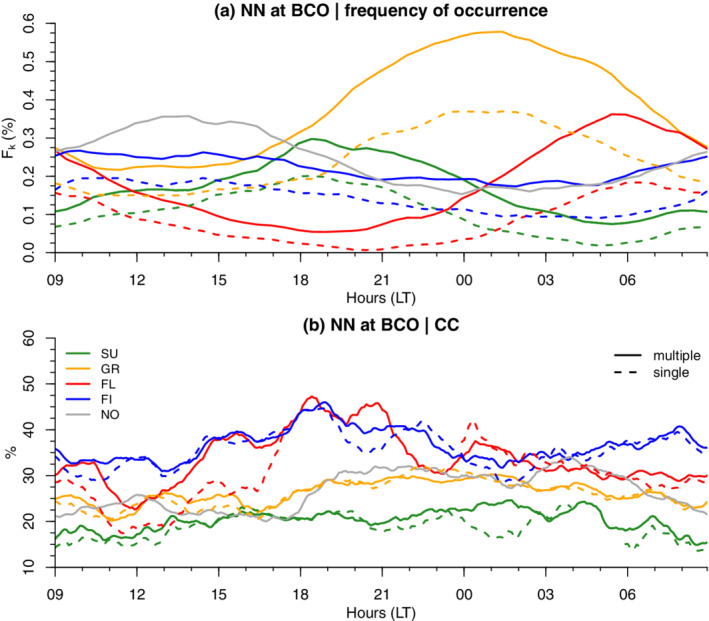
Daily cycle of (a) pattern frequency ($f_{k}$) and (b) BCO radar‐derived cloud cover ($CC_{k}$) when NN‐detected patterns overlap the site BCO. The solid lines refer to the case for which we allow multiple pattern occurrences over BCO and the dashed lines refer to the case for which only one pattern is detected at a given timestep

Note that while in the formulation above, the domain area corresponds to the sum of all NN‐pattern areas (including unclassified patterns), in practice here $a_{\text{dom}}$ tends to overestimate the actual domain size due to the overlaps between rectangles of different patterns. The difference between $a_{\text{dom}}$ and the actual domain size is somewhat proportional to the number of detected patterns, ranging between 15% in the afternoon and 30% at night‐time (Figure A3). While this difference seems significant, tests on the accuracy of pattern detection have shown that the interpretation of our results is not sensitive to these overlaps (Figures A3 and A4). Our findings also remain consistent when we discard multiple pattern occurrences at one timestep and location (Section 2.2 and Figure A5).

In addition to the “domain‐mean” pattern‐related cloud covers, we also consider the GOES‐16 ABI cloud cover for those pattern rectangles that overlap specific locations on the domain (i.e., the location of the instrumented sites described in the following section). In doing this, we similarly weight the pattern cloud cover by its spatial coverage, except for the 'No' category (which is not a distinct class, and thus does not have a delineated area around the site location).

### 2.2 Ground‐based remote‐sensing and insitu data at BCO and NTAS

Following Vial *et al*., (2019), we use ground‐based radar, ceilometer, and Micro Rain Radar (MRR) measurements from the Barbados Cloud Observatory (BCO), which is located at the most windward tip of Barbados at 59.48°W, 13.15°N and samples undisturbed trade‐wind conditions (Nuijens *et al*., 2014 and Stevens *et al*., 2016 give detailed descriptions of the BCO and its instrumentation). The cloud and rain statistics are aggregated into 5‐min averages. Periods with a radar signal between 4 and 8 km, including the hours before and after, are discarded to limit our analysis to shallow convection.

The mean rain rate is derived from MRR data at 325 m above ground (the lowermost level with reliable data). The MRR is also used to compute a rain flag, which is set to 1 when rain rates greater than 0.05 mm·hr${\;}^{-1}$ are measured in at least five range gates in the lowest 3 km (following Nuijens *et al*., 2014).

The vertical distributions of hydrometeors (i.e., cloud and rain droplets) and clouds are derived from a 35.5 GHz (Ka‐Band) Doppler cloud radar. The hydrometeor mask is derived using a threshold of −50 dBZ on the equivalent radar reflectivity $Z_{e}$ (a 10 dBZ lower threshold than used in Vial *et al*., 2019 to increase the sensitivity to smaller clouds). Cloud fraction profiles are obtained from the hydrometeor mask by discarding periods of rain: when the ceilometer does not detect a cloud base (due to strong rain) or when the MRR rain flag is 1. Moreover, radar signals below the ceilometer‐detected cloud‐base height are set to 0 in the cloud fraction profiles. Periods when neither the ceilometer nor the MRR are running are also discarded.

The (rain‐corrected) radar cloud profiles are also used to derive the total cloud cover, as well as the contribution to the total cloud cover from three distinct categories of clouds: (a) shallow cumuli with cloud base (CB) below 1 km and cloud top (CT) below 1.3 km, (b) deeper cumuli with CB <1 km and CT >1.3 km, and (c) clouds aloft with CB >1 km. This decomposition is slightly different than the more commonly used decomposition from Nuijens *et al*., 2014, as the use of radar rather than ceilometer data allows us to further decompose the LCL cloud category into two sub‐categories according to the cloud‐top height. The decomposition used here is similar to the one applied to model data in Vial *et al*., 2019 (details in their Appendix A).

Note that the rain‐correction applied will lead to an underestimation of total cloud cover, as periods of rain are usually also periods of cloudiness. Because rain is most frequent during night‐time (Nuijens *et al*., 2009; Vial *et al*., 2019) and because the rain frequency also depends on the patterns (i.e., Fish is the rainiest, followed by Flowers and Gravel, as found in Schulz *et al*., 2021), we tested that this underestimation does not bias our results by comparing the daily cycles of the total cloud cover and the total hydrometeor cover. The hydrometeor cover includes both cloud and rain droplets and thus overestimates cloud cover (being on average ∼10% larger for Sugar, ∼15% larger for Gravel and Flowers, and ∼20% larger for Fish). However, we find the daily cycles of total cloud cover and total hydrometeor cover to be very similar for all patterns (compare the black and grey curves on the top‐right panels of Figure 7), and thus conclude that the underestimation of cloud cover due to the rain‐correction does not bias our results.

We also use measurements of sea surface temperature (SST) and near‐surface wind speed from the Northwest Tropical Atlantic Station (NTAS) open ocean surface mooring at 51.02°W, 14.82°N. NTAS measurements have a temporal sampling rate of 1 min. Wind data are collected at about 3 m above sea level and SSTs at 1 m depth. To directly compare the buoy observations of wind speed with ERA5 estimates (Section 2.3), we adjust the 3 m wind to conform to the reference height of 10 m, using the simple power‐law wind profile ($u_{2}=u_{1}{\left(z_{2}/z_{1}\right)}^{0.11}$ – where $u_{2}$ is the wind speed at the reference 10 m height ($z_{2}$), and $u_{1}$ the wind speed measured at height $z_{1}$ (= 3.4 m)), which was shown to be a good approximation for use over the ocean, where near‐neutral stability conditions prevail (Hsu *et al*., 1994). This adjustment implies an increase of the wind speed of about 1 m·s${\;}^{-1}$ between 3 and 10 m.

In a similar way as described in Section 2.1, we construct pattern‐related composites for the BCO and NTAS sites, but we only select the NN‐detected patterns overlapping the location of these instrumented sites. The compositing is instantaneous in the sense that we average all measurements within ± 15 min around the classification time.

As explained above, several pattern rectangles can be detected at one location, potentially introducing a bias in the composite when different patterns occur at the same time. That said, we show in Figure A5 that discarding the timesteps when multiple labels occur at BCO does not affect the results, but does reduce significantly the sample size of our composites. We therefore keep all detected patterns at the site locations to construct the pattern‐related composites.

### 2.3 The large‐scale environment from ERA5 reanalysis

The ERA5 reanalysis is based on the Integrated Forecasting System (IFS) Cy41r2, operational since 2016. It provides hourly estimates of atmospheric variables at a horizontal resolution of 31 km (0.25° or TL639) and 137 vertical levels from the surface to 0.01 hPa (Hersbach *et al*., 2020).

We here use hourly output for the 10 m wind speed and the lower tropospheric stability (LTS, defined in Klein and Hartmann, 1993 as $\theta_{700}-\theta_{1000}$, where θ is the potential temperature in Kelvin) over the NN classification domain (45°–66°W, 9.3°–23.3°N), in order to explore how the daily cycles of cloudiness and mesoscale patterns relate to these large‐scale environmental factors that are known to play a role in the variability of trade‐wind cloudiness and organization at longer time‐scales (Brueck *et al*., 2015; Nuijens *et al*., 2015; Bony *et al*., 2020).

Following the approach described in Section 2.1, we construct the pattern‐related composites for the near‐surface wind speed ($U_{k}$) and LTS ($LTS_{k}$) and sample their daily cycle. The relationship between the large‐scale environment (including U and LTS) and the mesoscale patterns of organization was first explored in observations at the day‐to‐day and interannual time‐scales using the $I_{\text{org}}/S$ pattern classification method (Bony *et al*., 2020). Here, we extend the analysis by considering the daily time‐scale and patterns detected with the NN approach. Note also that using the $I_{\text{org}}/S$ method, combined with hourly estimates of the environmental conditions, the sample size is too small to see a robust signal on the daily time‐scale.

## 3 DAILY CHANGES IN THE OCCURRENCE OF MESOSCALE PATTERNS OF ORGANIZATION

The first question we address here is whether the observed occurrence of mesoscale patterns of shallow convection varies at the daily time‐scale.

Figure 3a,b shows that, whatever the method we use to classify mesoscale organization ($I_{\text{org}}/S$ or NN), the frequency of occurrence of all forms of organization exhibits a pronounced daily cycle with distinct 24 hr phasing. The patterns Fish and Sugar preferentially occur during daytime, and as such can be viewed as daytime patterns, while Gravel and Flowers appear rather as night‐time patterns. More specifically, the frequency of Fish patterns increases in the morning and reaches a maximum at 1200 hr; the peak in the Sugar pattern frequency is shifted towards the afternoon, at 1400 hr for $I_{\text{org}}/S$ and 1700 hr for NN (Table 1); Gravel increases during the afternoon and peaks at midnight; and the Flowers population grows soon after sunset until reaching a peak at the end of the night between 0300 and 0600 hr depending on the method.

The amplitude of these daily cycles is substantial, with a minimum of 20% relative to the daily mean for the NN‐detected Fish, between 35 and 60% for NN‐detected Gravel, Sugar and Flowers, and more than 100% daily variation for the $I_{\text{org}}/S$‐detected Sugar and Flowers patterns (Table 1).

When using the NN approach, the frequency of pattern occurrence depends both on the temporal frequency (Figure 3c) and spatial coverage of patterns (Figure 3d). These two attributes of pattern occurrence exhibit very similar diurnal phasings, which means that when the NN detects more of a given pattern, it also extends over a larger area and viceversa. However, the daily variability in the occurrence of patterns (Figure 3b) appears to be more strongly driven by their temporal frequencies – whose variations range between 10 and 45% with respect to daily means – than their spatial coverage, which varies between 5 and 15% (relative to daily means) among the different patterns.

Gravel appears to be the most frequent pattern and on average covers larger areas of the domain (more than 50% of the domain area on average). The Flowers pattern also covers about half of the domain on average, but with a very pronounced daily cycle in its temporal frequency – it is the least frequently observed pattern around sunset, as well as one of the most frequent patterns at night. Sugar and Fish are the least spatially extended patterns (about 30 and 40% of the domain, respectively), but they can be frequently observed, especially during daytime (about 60% of the time at 1200 and 1700 hr, respectively).

Similar daily phasings as on the entire domain are also found when looking at the surface sites BCO and NTAS independently. However, the comparison between BCO and NTAS (which lies 8° east of BCO) reveals a geographical dependency in pattern occurrence, especially regarding the daily means and amplitudes of the daily cycles in the temporal frequency of patterns (Figure 3e). Overall, the frequency of detected patterns is systematically higher at NTAS than at BCO, while the frequency of unclassified patterns is greater at BCO. Note that larger differences were found, especially for the unclassified category, by applying the NN algorithm on a 5°‐eastward shifted domain (not shown). This was due to a lower pattern detection at BCO, which lay closer to the edge of the domain. With the present domain, however, BCO and NTAS are equally distant from the edge of the domain (about 6°), thus we expect the differences between the two sites to be real.

In particular, the differences for the Gravel and the Flowers patterns, which are the most important throughtout the 24 hr daily cycle, might reflect an east–west gradient in the frequency of occurrence of these patterns, with a greater occurrence on the east due to stronger easterlies (not shown). This is consistent with the findings in Bony *et al*., (2020) who show that the Gravel and Flowers patterns mostly occur in conditions of stronger near‐surface wind speed. The frequencies of the Fish patterns are fairly similar at BCO and NTAS, which can be expected given the large‐scale characteristic of this pattern which, moreover, is most often oriented along the east–west direction (Schulz *et al*., 2021; also Figure 9). Finally, we note also that the daily amplitude of the Sugar pattern is higher at NTAS than at BCO due to a much higher occurrence frequency during daytime.

## 4 DEPENDENCE OF THE DAILY CYCLE OF TRADE‐WIND CUMULI AND PRECIPITATION ON MESOSCALE PATTERNS OF ORGANIZATION

### 4.1 Mesoscale pattern signatures on the daily cycle of GOES‐16 ABI cloud cover

**FIGURE 4:**
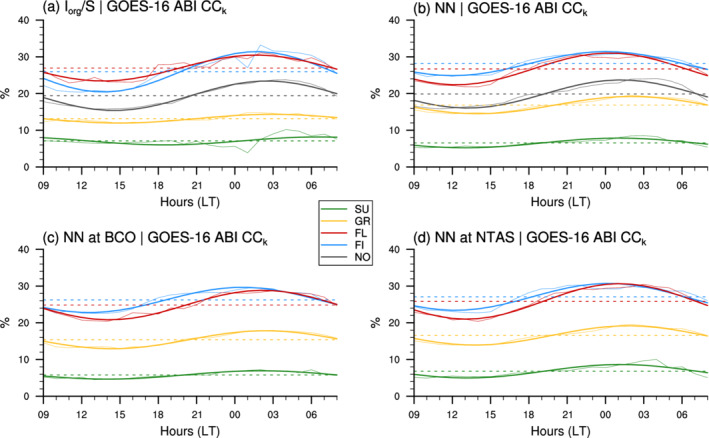
Daily cycles and first harmonics of patterns cloud cover ($CC_{k}$) derived from GOES‐16 ABI using (a) $I_{\text{org}}/S$ classified patterns, (b) NN‐detected patterns over the entire domain, (c) NN‐detected patterns over BCO and (d) NN‐detected patterns over NTAS. The dashed lines represent the daily‐mean cloud covers. Note that the NO category does not appear in (c, d) as there is no delineated area over which we can compute a cloud cover. The colour code is as in Figure 3

Figure 4 shows the averaged daily cycle of GOES‐16 ABI cloud cover associated with the different patterns ($CC_{k}$) detected over the entire classification domains (panels a and b) and over the sites at BCO (c) and NTAS (d). The similarity between all four panels is salient, and suggests that the daily cycle in cloudiness is overall independent of the classification method, of the patterns themselves, and of the geographical location.

The weak dependency of the daily phase of cloud cover on NN patterns is particularly striking, especially so for the large domain (Figure 4b): cloudiness always minimizes in the afternoon (between 1300 and 1600 hr) and maximizes at night‐time (between 0000 and 0400 hr), consistent with Vial *et al*., (2019) and the 3‐year winter‐time climatological daily cycle in cloud cover calculated here (cf. black lines in Figure 6 below). The exception is for Sugar identified with $I_{\text{org}}/S$, which shows a phase shift of about 5 hr, although the robustness of this daily cycle might be questioned given the small number of Sugar cases identified with $I_{\text{org}}/S$ at night‐time (Figure 3a).

From a more quantitative point of view, the daily mean and amplitude in cloud cover are, to some extent, dependent on pattern, and more precisely, on the mean size of cloud objects (S): patterns with small cloud entities (Sugar and Gravel), tend to have a smaller daily mean and amplitude in cloud cover than the Flowers and Fish patterns, which have much larger cloud structures and a larger fraction of stratiform cloudiness near the inversion that is particularly sensitive to the daily cycle (Vial *et al*., 2019). Nevertheless, the daily variability in cloud cover for a given pattern remains small compared to the differences in daily‐mean cloud cover between the patterns. The cloud cover varies by at least a factor of two across the different patterns (consistent with Bony *et al*., 2020), while the daily variations range between 10 and 20% relative to daily means.

Note that Bony *et al*., (2020) found a higher cloud cover (from MODIS cloud products) for the Flowers pattern than for the Fish pattern identified with $I_{\text{org}}/S$, which is consistent with our daytime estimates (Figure 4a), given that only daytime measurements of MODIS over the North Atlantic trade‐wind region (mid‐morning for Terra and early afternoon for Aqua) were used. Nevertheless, it should also be noted that our GOES‐16 ABI estimates of cloud cover are overall lower than MODIS estimates (cf. Bony *et al*., 2020), presumably because the $T_{b}$ cloud mask and lower resolution of GOES‐16 ABI prevent detection of the smallest clouds (a point that is further discussed at the end of Section 4.2).

**FIGURE 5:**
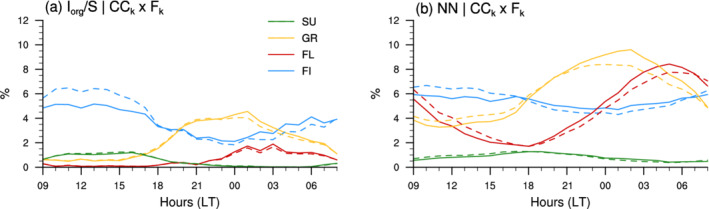
Daily cycle in the effective pattern cloud covers ($CC_{k}\times F_{k}$, solid lines) from GOES‐16 ABI using (a) $I_{\text{org}}/S$ classified patterns and (b) NN‐detected patterns over the entire domain. The dashed lines represent the product $CC_{k}\times F_{k}$ but with $CC_{k}$ fixed to the daily mean. The colour code is as in Figure 3

Overall, our results suggest that the mesoscale patterns of cloud organization constitute a fairly robust constraint on cloud cover, and that the dependence of the daily cycle on mesoscale organization is essentially due to the daily changes in pattern frequency of occurrence. This also means that, knowing the daily variation in pattern occurrence and the mean cloud cover for a given pattern, we can recover to a large degree the daily cycle in the effective cloud cover of the different patterns (Figure 5).

### 4.2 Contribution of mesoscale patterns to the daily cycle of cloud cover

**FIGURE 6:**
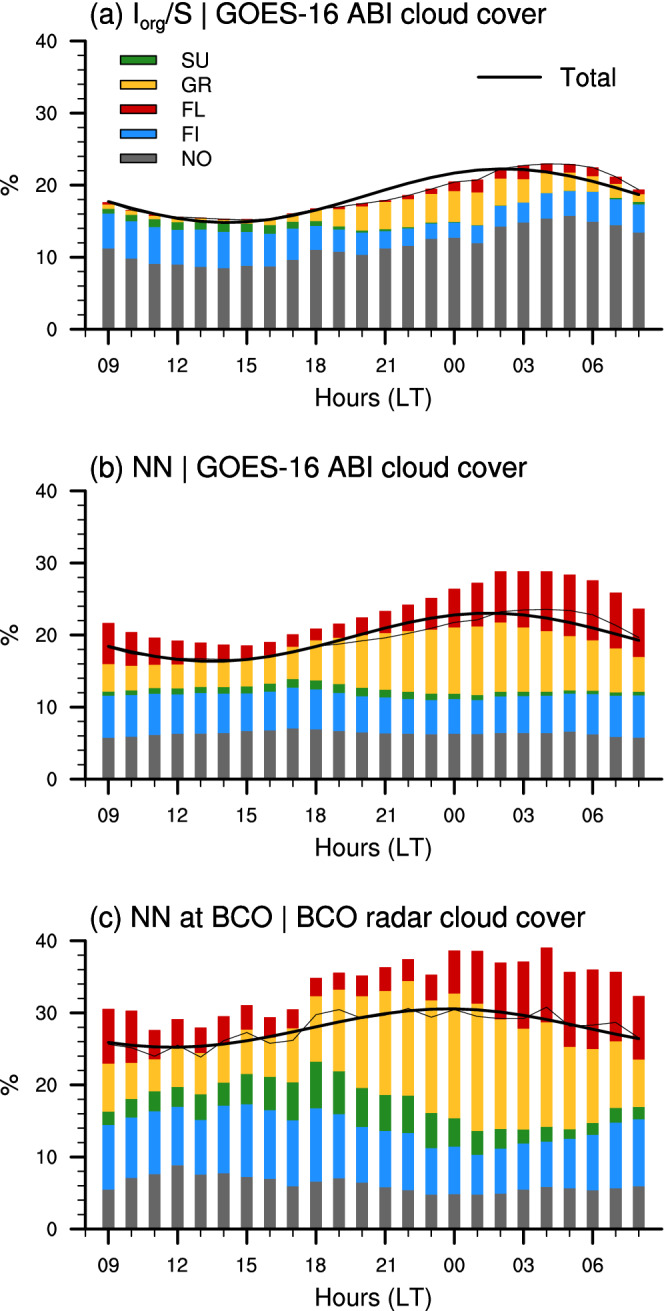
Daily cycle of total cloud cover (black thin line, with the first harmonics superimposed in thicker line) and relative contributions of the different mesoscale patterns of cloud organization ($CC_{k}\times F_{k}$): Sugar (green), Gravel (yellow), Flowers (red), Fish (blue) and No classification (grey). In (a, b) the pattern cloud covers are derived with GOES‐16 ABI $T_{b}$ cloud mask on (a) the 10${\;}^{\circ }\times 10^{\circ }$ $I_{\text{org}}/S$ domain and (b) the NN‐detected patterns within the entire 14${\;}^{\circ }\times 21^{\circ }$ classification domain. (c) is as (a, b) but the cloud covers are diagnosed from the radar at BCO using NN‐detected patterns overlapping the BCO site

Combining the daily cycles in $F_{k}$ (Section 3) and $CC_{k}$ (Section 4.1) into the product $CC_{k}\times F_{k}$ allows us to quantify more explicitely the relative contribution of mesoscale patterns to the daily cycle in total cloud cover. We present this “effective pattern cloud cover decomposition” in Figure 6 for GOES‐16 ABI cloudiness and detected patterns over the entire classification domains (panels a and b), as well as for the radar cloud cover at BCO and the NN‐patterns overlapping the BCO site (panel c). Recall that, when using the NN‐patterns, the sum of all contributions is greater than the total cloud cover (black lines) because of the overlaps between multiple label occurrences (Section 2.1.1).

Overall, the contribution to total cloudiness from the four defined patterns is greater at night – when the total cloud cover is a maximum – than during the day. However, the extent to which the patterns explain the total cloudiness strongly depends on the classification method. When using $I_{\text{org}}/S$, about 60% of total cloudiness is explained by the No category throughout the 24 hr cycle (Figure 6a). This percentage reflects the frequency of occurrence of the No category (Figure 3a) which is set by the classification criteria of patterns (Section 2.1.1). With the NN method, only about 30% of the cloud cover is due to the No category, and the daily cycle for this contribution remains weak (Figures 6b,c) owing to the opposite diurnal phasing of $F_{\text{No}}$ and $CC_{\text{No}}$ (compare for instance Figures 3b and 4b).

However, the contribution of the No category to the overall daily cycle in cloud cover deserves some more discussion. It is notable that adding the percentage of cloud cover due to the overlaps between NN patterns (i.e., the difference between the sum of all contributions and the actual total cloud cover in Figure 6b) and the percentage of $CC_{\text{No}}\times F_{\text{No}}$ (the grey area in Figure 6b), leads to a similar contribution of *unclear* patterns as that of the No category for $I_{\text{org}}/S$. This further supports the coherence between these two intrinsically different methods. Moreover, it suggests that using the NN method, we can unravel the contribution to total cloud cover due to forms of cloud organization that are somehow related to the four predefined patterns (i.e., the contribution from overlaps) and that due to organization forms that are not related to the predefined patterns (i.e., contribution from the No category). Therefore, we argue here that the most likely contribution of unclassified forms of organization to total cloud cover is about 30% (the percentage given by the NN method) – and thus that the largest extent of cloud cover can be explained by these four forms of mesoscale cloud patterns, as follows.

The Fish pattern is the most important contribution to the afternoon cloudiness regardless of the classification method (Figure 6a,b). This is partly because this pattern occurs more frequently during daylight hours (especially when using $I_{\text{org}}/S$), but also because the other patterns are less frequent and therefore contribute relatively little to cloudiness at this time. Actually, when using the NN method, the effective contribution of the Fish pattern to the daily cycle in cloud cover tends to be quite small (Figure 6b,c), because of opposing phases between $F_{\text{FI}}$ and $CC_{\text{FI}}$ (Figure 5b) and relatively weak daily amplitudes in $F_{\text{FI}}$ (Table 1 and Figure 3). Note that the Fish pattern is often associated with a synoptic disturbance that persists for several days, continuously forced by a convergence line (Aemisegger *et al*., 2021; Schulz *et al*., 2021). This may thus explain the small daily cycle in the occurrence frequency of the Fish patterns.

When using the NN method, Gravel and Flowers are the dominant contributions at night‐time, both over the entire classification domain and at BCO. Gravel explains about 45% of the total cloud cover around midnight, while the Flowers contribution maximizes just before sunrise with values ranging between 30% (at BCO) and 40% (over the large domain). A similar 24 hr phasing is observed for these two patterns when using $I_{\text{org}}/S$, although their contribution to total cloudiness remains small (Figure 6a).

The case of the Sugar pattern is interesting because it can occur quite frequently (Figures 3c,e) but its spatial extent is relatively small (Figure 3d). Consequently, its contribution to total cloudiness appears much larger at BCO (Figure 6c) than over the large domain (Figure 6b). Moreover, the daily phases of $F_{\text{SU}}$ and $CC_{\text{SU}}$ are opposed (Figures 3b and 4b), which reduces the effective contribution of this pattern to the daily cycle of total cloudiness. With $I_{\text{org}}/S$, Sugar is the pattern that contributes the least to the total cloud cover and its daily cycle.

Overall, these results reveal that unclassified and Fish patterns are the most important for daytime cloudiness, while Gravel and Flowers contribute most to night‐time cloudiness. The contribution from the Sugar pattern, although never dominant, maximizes around sunset (with NN) and can be more important when viewed locally than at large‐scale.

Finally, we draw attention to the three different cloud cover estimates in Figure 6 (black lines). We note, in particular, that the cloud cover from the radar at BCO is about 3 to 10% larger than the satellite‐based estimates over the large domain. This difference is even larger when using the ceilometer‐based cloud cover (not shown). This difference, which has also been reported in previous studies comparing BCO data with other satellite‐based products (Nuijens *et al*., 2015; Vial *et al*., 2019), is likely due to different capabilities of the instruments to measure low‐level clouds – the BCO radar or ceilometer being much more sensitive to low‐level cumuli than the GOES‐16 ABI infrared channel. Moreover, the $T_{b}$ cloud mask is defined such as to exclude some of the shallowest clouds, in particular those with a cloud‐top height below 1 km (Bony *et al*., 2020). So we might expect the difference in the cloud cover estimates (between GOES‐16 ABI and the BCO radar or ceilometer) to be particularly pronounced for the Sugar pattern which essentially consists of clouds with little vertical extent above the LCL (Schulz *et al*., 2021). Although this difference is indeed slightly larger for the Sugar pattern, it remains, nevertheless, of the same order of magnitude regardless of the pattern (compare the different pattern‐related values of $CC_{k}$ in Table 1). This could be explained based on findings from Schulz *et al*., (2021) showing that the cloudiness near the LCL does not vary substantially from pattern to pattern, and therefore the difference between the BCO and satellite‐based estimates should also remain relatively similar from pattern to pattern.

### 4.3 Mesoscale pattern signatures on the daily cycle of clouds and precipitation at BCO

**FIGURE 7:**
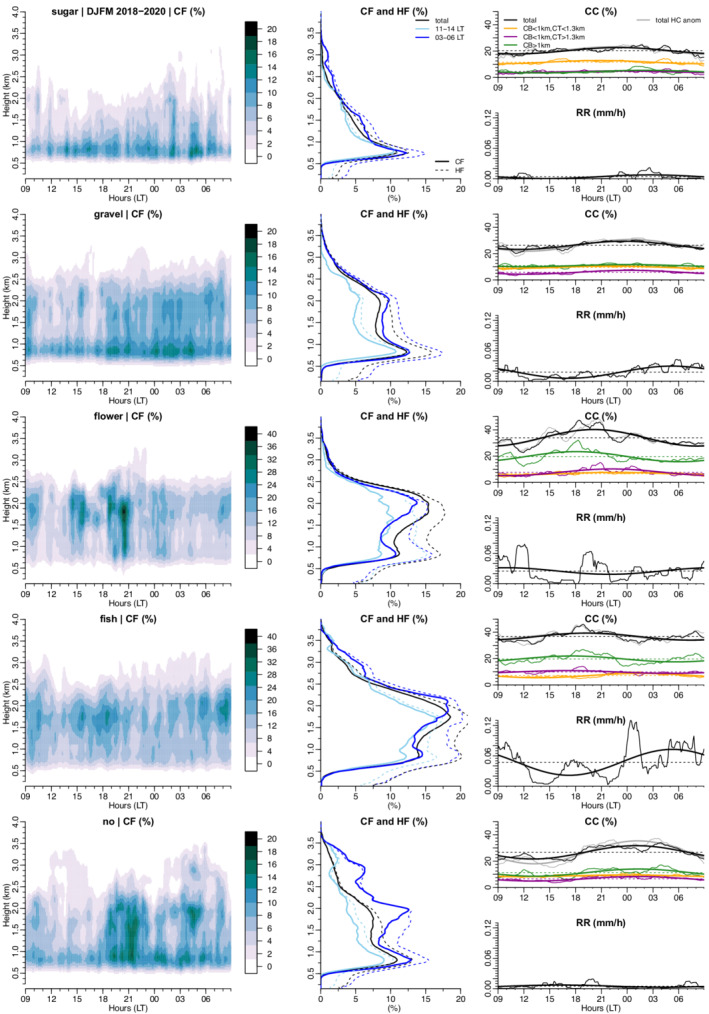
Pattern‐related daily cycle of cloudiness and precipitation from the Barbados Cloud Observatory for (top‐to‐bottom) Sugar, Gravel, Flowers, Fish and No patterns. Left panels: rain‐corrected cloud fraction derived from the radar. Middle panels: profiles at selected times for the rain‐corrected (cloud) and hydrometeor (cloud and rain droplets) fraction derived from the radar. Right panels: (top) radar‐derived cloud covers and (bottom) rain rate derived from the Micro Rain Radar (MRR). In the right panels, the thin solid lines represent the actual data, the thicker lines are the first harmonics, and the thin dotted horizontal lines are the daily means. Also shown in the top right panels (grey curve) is the daily cycle in total hydrometeor cover (HC), with the difference in the daily means between HC and CC removed (for ease of readability in the figure). The daily means in HC are 31.5% for Sugar, 40.6% for Gravel, 49.8% for Flowers, 57.4% for Fish and 46.3% for No

We here take advantage of the BCO dataset (Section 2.2) to characterize further the different cloud and precipitation properties associated with each pattern of organization. Schulz *et al*., (2021) provides a detailed description of the mean structure of clouds and of the convective boundary layer for each pattern. Here, we focus more on the daily evolution of precipitation and of the vertical distribution of cloudiness (Figure 7). We show that, even if the daily cycle of the overall cloud cover ($CC_{k}$) is very similar among the different patterns (Section 4.1), each organization pattern appears with its own daily cycle of shallow convection:

- The Sugar pattern essentially consists of small non‐precipitating cumuli with a cloud‐base height close to the LCL (below 1 km) and weak vertical extent during daylight hours. Near sunset, the overall cloud cover starts to increase due to a blooming of slightly deeper clouds reaching the upper cloud layer (above 1.3 km) between 1900 and 0200 hr. The proportion of shallower clouds (i.e., CT <1.3 km) is dominant and remains fairly constant throughout the day. However, despite this overall cloud deepening at night, the precipitation rates measured at the surface remain low throughout the day.
- For the Gravel pattern, clouds overall reach higher levels in the cloud layer, with about 2/3 of the cloud population having their top above 1.3 km. The daily cycle of the total cloud cover is mainly driven by the population of thicker clouds (CB <1 km and CT >1.3 km), which increases in the afternoon and maximizes at 0000 hr. The cloud cover from both the very shallow clouds (CB <1 km and CT < 1.3 km) and clouds aloft (CB >1 km) remains roughly constant throughout the day. Interestingly, the precipitation peak is delayed by about 6 hr with respect to the maximum in cloud cover. Seifert and Heus (2013) noted a similar feature in large‐eddy simulations of shallow convection with cold‐pool organization (cf. their figure 2). One reason could be that there is, first, a reduction of rainfall due to evaporation below cloud‐base and that, later, the generated cold pools created a moister environment allowing more rainfall at the surface. Further investigation is needed to verify the robustness of this time shift between cloudiness and precipitation and to provide an explanation for it.
- The Flowers pattern has to be interpreted with caution as the number of detected patterns is small over BCO, especially between 1800 and 2100 hr (Figure 3), at times when the peak in cloudiness is observed (Figure 7). Nevertheless, we find that about 80% of the cloud cover is explained by clouds with cloud‐top height above 1.3 km, and the cloud fraction near the inversion tends to be more pronounced at late night hours. The daily cycle in precipitation seems weak and local maxima are not always correlated with peaks in cloudiness (for instance at 1200 hr) – a feature that could also be explained by the presence of cold pools as for the Gravel case.
- For the Fish pattern, the relative contributions of shallow and deeper clouds are similar to the Flowers pattern. The daily variability in CC exhibits two local maxima, around sunset and at early morning hours, but with an overall tendency (given by the first harmonic) for a daily maximum in cloudiness around sunset. The vertical cloud fraction profiles reveal that the night‐time inversion and clouds can reach higher levels than during the day. The precipitation daily cycle given by the first harmonic is similar to the Gravel pattern, albeit with more pronounced variability.
- The unclassified cloud scenes (the “No” category) might be the most variable on the daily time‐scale. The cloud profile is fairly similar to that of the Sugar pattern during the day, whereas at night it appears as a mixture between the Gravel and the Fish patterns (with both a strong inversion near 2 km and a significant cloud fraction above). Despite the night‐time cloud deepening, surface rainfall remains quite low throughout the day. However, there is a small difference in the amplitude of the daily cycle between the total hydrometeor cover and the total cloud cover (of about 3% – between the grey and black curves in the top‐right panel), which suggests a night‐time enhancement of clouds with precipitation at higher levels.

**FIGURE A6:**
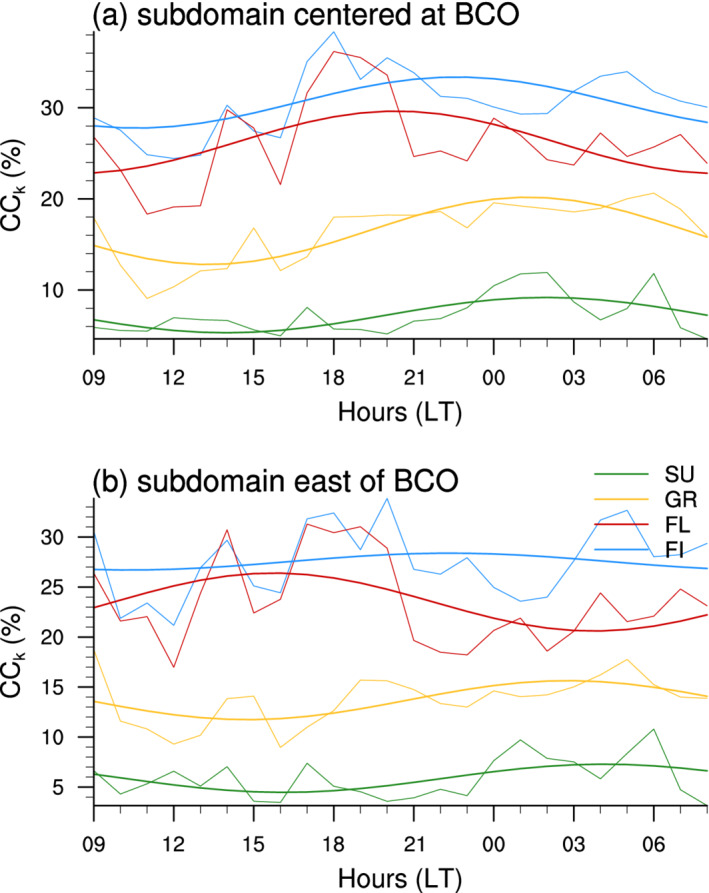
Daily cycle of pattern cloud covers diagnosed from GOES‐16 ABI for patterns overlapping BCO. The cloud covers are calculated over the part of the patterns that is contained in a 20×20 km${\;}^{2}$ sub‐domain (a) centred at BCO and (b) 10 km east of BCO

One notable feature worth mentioning is the different 24 hr phasing in pattern‐related cloud cover at BCO whether the cloud cover is derived using GOES‐16 ABI (Figure 4c) or BCO remote‐sensing instruments (Figure 7). When using the BCO measurements, the peak in cloud cover is systematically earlier in time than when GOES‐16 ABI retrievals are used (with a phase shift of several hours – between 2 and 7 hr – depending on the pattern; Table 1). The time difference in the solar forcing between the western and eastern boundary of the NN classification domain is at most 1 hr 24 min (4 min for every degree longitude), which cannot explain the aforementioned time shifts. However, one reason for these differences could be related to sampling: a small number of detected patterns at BCO (e.g., Flowers at 1800 hr) and/or a too small temporal averaging for a given pattern to capture the averaged properties of the pattern at a given time. For example, given the large size of Flowers (∼100 km or more), they can take several hours to cross entirely over BCO (figure 7 in Stevens *et al*., 2020). Complementary tests of the influence of spatial/temporal scales on our results support this explanation (Figure A6).

## 5 THE ROLE OF ENVIRONMENTAL FACTORS

Two related questions that can be asked now are what controls, on the one hand, the daily variability of pattern frequency and, on the other hand, the constancy in the night‐time peak of cloudiness regardless of the pattern. We shed light on these questions by diagnosing the daily cycle of some of the environmental factors that are known to be either determinant for pattern occurrence (Bony *et al*., 2020) and/or controlling factors of winter trade cloudiness at time‐scales longer than a day (Brueck *et al*., 2015; Nuijens *et al*., 2015). In those studies, the near‐surface wind speed and LTS appear to be the most influential factors on the day‐to‐day and interannual time‐scales. We thus consider those two variables, as well as the SST, which is known to be an important ingredient for the daily cycle of convection when the near‐surface wind speed is weak (Bellenger *et al*., 2010; Ruppert and Johnson, 2016).

**FIGURE 8:**
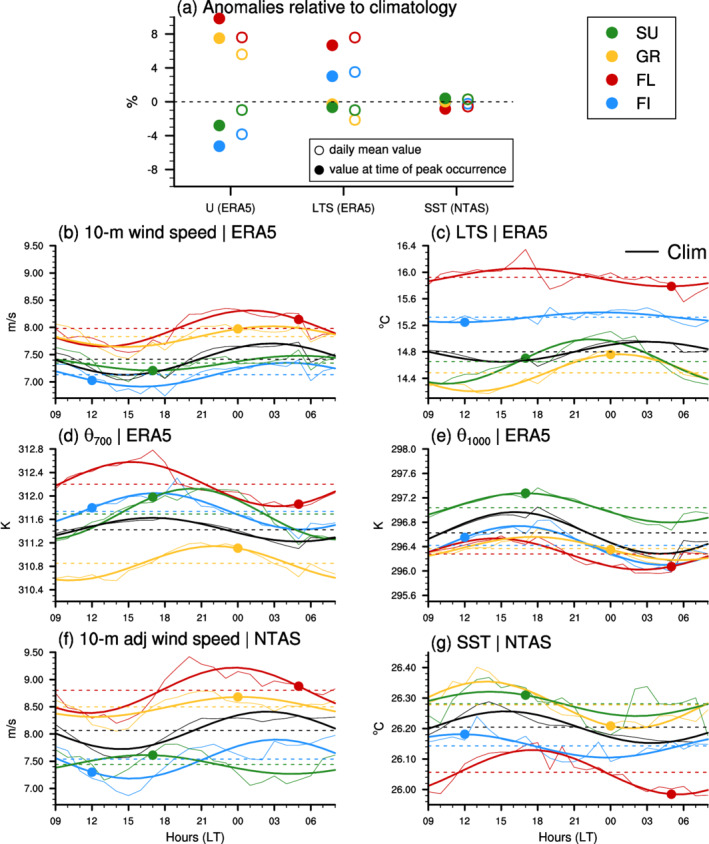
(a) Fractional anomalies (with respect to climatological mean) of pattern‐related large‐scale 10 m wind speed (U) and LTS from ERA5 and local SST from NTAS measurements. The empty circles represent the anomalies based on the daily‐mean U, LTS and SST, whereas the filled circles represent the anomalies at the time of the peak occurrence of the specific patterns (which is indicated by the filled markers in (b–g). (b–e) show pattern‐related daily cycle of U, LTS, $\theta_{700}$ and $\theta_{1000}$ sampled over the large NN classification domain when using ERA5 data. (f, g) show pattern‐related daily cycle of U and SST sampled over the NTAS site and using NTAS measurements. The first harmonics are shown by the thicker line. Note that the 3 m wind speed measured at NTAS has been adjusted to conform to the reference 10 m height (Section 2.2). The colour code for the patterns is indicated in the legend, and the black line represents the climatological mean (DJFM 2018–2020)

The results presented in Figure 8 show that these three variables exhibit a daily cycle, with distinct phasings and amplitudes depending on the variable itself, on the pattern, and on the dataset that is considered.

### 5.1 Relation to the daily cycle in pattern frequency

For daily means in large‐scale wind speed and LTS, our findings are consistent with Bony *et al*., (2020): the large‐scale environment tends to be less stable with weak winds for Sugar, less stable with strong winds for Gravel, more stable with strong winds for Flowers, more stable with weak winds for Fish (Figure 8a, empty circles). Here, we show that this holds at any time of the day, and that the daily cycle in wind speed amplifies the relationship between wind speed and pattern occurrence (Figure 8a, filled circles). The large‐scale wind tends to be stronger at night regardless of the pattern (Figure 8b), and thus it discriminates the occurrence of the organization patterns in the same way at sub‐daily, daily and interannual time‐scales: Gravel and Flowers occur mostly at night when the wind is stronger, while Fish and Sugar occur mostly during daytime when the wind is weaker. However, it is worth noting that this relationship can be different depending on whether the wind speed is diagnosed at large‐scale (here, with ERA5 over the large NN classification domain in Figure 8b) or locally (e.g., at NTAS in Figure 8f). Indeed, the overall increased variability in the pattern‐related daily cycles in wind speed at NTAS could explain the difference for the Sugar pattern (compare the thin lines between Figure 8b and f).

The daily cycle in LTS can be different from one pattern to another (Figure 8c), and the relationship between LTS and pattern occurrence at daily time‐scale is opposite to that found at longer time‐scales: at times of maximum occurrence of Fish and Flowers patterns, the environment is the least stable of the day, and at times of maximum occurrence of Gravel and Sugar patterns, the environment tends to be more stable compared to the pattern‐related daily‐mean LTS (Figure 8a).

The SSTs tend to be lower for the Flowers pattern and higher for the Sugar pattern (as in Bony *et al*., 2020), but overall this variable does not significantly explain the variability in pattern occurrence on the day‐to‐day or daily time‐scales (Figure 8a).

Therefore, the near‐surface wind speed is here the factor that explains best the daily variability in pattern occurrence; it discriminates between the daytime and night‐time patterns.

### 5.2 Relation to the constancy of the night‐time peak of cloudiness

As mentioned earlier, the large‐scale wind is overall stronger at night whatever the pattern (Figure 8b), and thus correlates quite well with the daily cycle in pattern‐related cloudiness (Figure 4). This co‐variability between trade‐wind cloudiness and near‐surface wind speed has already been discussed in the context of slowly varying observations (Brueck *et al*., 2015; Nuijens *et al*., 2015) or in equilibrated large‐eddy simulations (Nuijens and Stevens, 2012). Here, we show that it happens on the daily time‐scale whatever the organization pattern in place, and could therefore constitute a basic ingredient of trade‐wind convection: as the winds reinforce, surface evaporation increases, providing the moisture that is needed for the clouds to grow deeper, which then helps increase the overall cloud cover. Surface winds, in turn, can be enhanced at night as radiative cooling destabilizes the boundary layer and strengthens the momentum transport by the shallow convection (Hourdin *et al*., 2015; Schlemmer *et al*., 2017).

The domain‐mean LTS has a small daily cycle with a maximum at night‐time – at times when the cloud cover is maximum (compare black line in Figures 6b and 8b). This result is somewhat expected given that large night‐time cloudiness is mainly related to the spreading of a stratiform cloud layer below the trade inversion (Vial *et al*., 2019), and that stratiform cloudiness is more likely to occur under stronger stability (Wood and Bretherton, 2006). Given that strong LTS is favoured by weak $\theta_{1000}$ and/or strong $\theta_{700}$ and that both $\theta_{1000}$ and $\theta_{700}$ exhibit a daily cycle with a minimum at night‐time, the night‐time maximum in LTS is then primarily due to the minimum in (near‐)surface warming.

Note, however, that the daily cycle in LTS is different from one pattern to another; this is related to a $\theta_{700}$ dependency, as the daily cycle in $\theta_{1000}$ is fairly similar for all patterns (Figures 8d,e). The Flowers pattern in particular is associated with less stable conditions during the night, owing to a large decrease in $\theta_{700}$. From these results, we hypothesize that, while more stable conditions can be more favourable to stratiform cloudiness at night, once the Flowers stratiform cloud layer are present they might produce locally less stable conditions, presumably through enhanced radiative cooling at cloud‐top (e.g., figure 6 of Albright *et al*., 2021). Case‐studies of the field campaign EUREC${\;}^{4}$A (Stevens *et al*., 2021) which took place windward of Barbados in January–February 2020 could provide new opportunities to further investigate how the cloud patterns impact the local environment.

Due to the sustained easterlies in this season, the daily cycle in SST is not expected to be strong and hence also is not expected to play a major role in the daily cycle of trade‐wind cumuli (Brill and Albrecht, 1982; Vial *et al*., 2019). Indeed, as shown in Figure 8e, the daily amplitudes of SSTs remain small, ranging between 0.1 and 0.2°C across the different patterns. Moreover, there is an overall tendency for higher SSTs during the day, at times when the cloud cover is minimum, revealing that the nocturnal increase in cloud cover is not forced by surface warming (a somewhat obvious fact).

The dependence of the SST daily cycles on the mesoscale patterns of organization and associated wind speed does not appear straightforward. Although there is an anticorrelation between the daily cycles of the wind speed and of the SST for the three‐winter climatology (black curves in Figures 8d,e), consistently with previous observational analyses over this area on long time‐scales (e.g., Xie, 2004), more diverse relationships are found at the daily time‐scale for the individual patterns. We note, for instance, a time shift of about 6 hr between the daily maximum in wind speed and the daily minimum in SST for the patterns Sugar and Flowers. Moreover, there does not seem to be a linear relationship between the daily‐mean wind speed and SST among the different patterns. These results therefore suggest that different factors (e.g., precipitation, upper ocean eddies) might affect the air–sea coupling on short time‐scales and depending on the mesoscale pattern of cloud organization.

## 6 CONCLUSIONS AND DISCUSSIONS

High‐frequency geostationary satellite observations over the tropical Atlantic ocean and ground‐based remote‐sensing measurements from the Barbados Cloud Observatory (BCO) are used to explore how the daily cycle of cloudiness in the winter trades depends on the spatial organization of shallow convection. We focus on the four prominent patterns of cloud organization of this region – Sugar, Gravel, Flowers and Fish – which have been characterized recently (Stevens *et al*., 2020). We apply two existing classification methods on 30 min infrared brightness temperatures from GOES‐16 ABI to sample the daily cycle of these four forms of organization: one based on a neural network (referred to as NN) and one based on the mean size and degree of clustering of segmented cloud objects (referred to as $I_{\text{org}}/S$). A fifth category is also considered for unclassified mesoscale cloud scenes. Although these two classification methods are quite different in nature, they both yield qualitatively similar results, which are summarized below:

1. All forms of mesoscale organization exhibit a pronounced daily cycle in their frequency of occurrence, with distinct phasing and amplitude. The patterns Fish and Sugar preferentially occur during daytime, with a frequency peak around noon for Fish and around sunset for Sugar. The patterns Gravel and Flowers occur more frequently during night‐time; Gravel maximizes around midnight and Flowers at early morning hours before sunrise. From a more quantitative point of view, the daily characteristics of pattern occurrence (mean, phase, amplitude) are somewhat dependent on the classification method and on the geographical location of the patterns. The dependence of pattern occurrence to the large‐scale environmental factors, such as the east–west gradient in near‐surface wind speed, can explain some of the geographical disparities.
2. The daily cycle in cloudiness for a given pattern is relatively weak compared to the differences in cloudiness between the patterns. It is also fairly independent of the pattern and its geographical location: any given pattern cloud cover is maximum at night‐time (between 0000 and 0300 hr) and minimum in the afternoon (between 1200 and 1500 hr).
3. As a result of points 1 and 2, the effective contribution of patterns to the daily cycle in total cloudiness is to a large extent mediated by the frequency of pattern occurrence. The Fish pattern, which mostly occurs during the day, explains about 30% of daytime cloud cover. The contribution to total cloud cover from the Sugar pattern is the most important around sunset, representing up to about 25% of total cloud cover at this time at BCO. Gravel is the dominant form of organization around midnight (explaining up to 45% of total cloudiness), and Flowers can contribute up to 40% at early morning hours before sunrise.
4. A significant contribution of total cloud cover is also associated with unclassified organization, especially during daytime, which happens to be at the time of minimum cloud cover. Nevertheless, and although the importance of the unclassified contribution depends on the classification method, we find that the mesoscale patterns of cloud organization can explain to a large extent the daily cycle in total cloud cover.

A more detailed analysis of the cloud vertical distribution and precipitation at BCO allows to connect our findings with the description of the daily cycle of shallow cumuli made in Vial *et al*., (2019). First, they showed that during daytime a population of very shallow clouds grows, reaches a peak at 1800–1900 hr, and decays until dawn. Here we demonstrate that this behaviour is associated with an increased occurrence frequency of the Sugar pattern towards sunset. Second, the overnight cloud deepening discussed in Vial *et al*., (2019) is here primarily connected to the increased occurrence of the Gravel pattern, and to a lesser extent, to an overall deepening of clouds embedded in the Sugar and Gravel patterns. Third, the dawn peak in cloud cover owing to the spreading of a stratiform cloud layer below the trade inversion (Vial *et al*., 2019) is here connected to a maximum occurrence of the Flowers pattern at this time of the day. It thus appears that the daily cycle in the occurrence of Sugar, Gravel and Flowers together may, to some extent, explain the evolution of trade‐wind cloudiness from the shallowest cumuli in late afternoon to the night‐time population of deeper cumuli. These insights raise the question of the autocorrelation time‐scale of individual patterns, and of the evolution from one pattern to another, which we leave for future investigation. In that respect, we expect the Fish pattern to be somewhat different, as it appears more strongly connected to non‐local synoptic‐scale disturbances persisting on time‐scales longer than a day (Aemisegger *et al*., 2021; Schulz *et al*., 2021). This is further supported here with a relatively weak daily cycle in the occurrence frequency of the Fish pattern.

The early morning peak in surface precipitation, identified in previous studies (Nuijens *et al*., 2009; Vial *et al*., 2019), is here associated with both the peak occurrence of the nocturnal patterns (Gravel and Flowers) and the enhanced rain rate at the end of the night for the *‘rainy’* patterns (Gravel, Flowers and Fish), regardless of their time of occurrence. Interestingly, for those three patterns (and especially for Gravel), the rain rate peak tends to succeed the cloud cover maximum by a few hours, which might be related to cold pools. This hypothesis should motivate further investigation to ascertain whether cold pools actually play a role in the phasing of rainfall.

Finally, we find that the large‐scale near‐surface wind speed can explain some of the geographical disparities in pattern frequency, it can also robustly discriminate between daytime and night‐time patterns, and it is fairly related to the daily cycle in pattern cloudiness regardless of the pattern in place. These insights, combined with findings from previous studies (Brueck *et al*., 2015; Nuijens *et al*., 2015; Bony *et al*., 2020), suggest that the strength of the trade winds are overall tightly connected to cloud amount and organization over a wide range of time‐scales from sub‐daily to inter‐annual.

## APPENDIX A

### A.1 Characterization of shallow cloud organization from the paired ($I_{\text{org}},S$) distributions

We consider the NN approach as more subjective (than the $I_{\text{org}}/S$ method) in the sense that the NN algorithm is trained from satellite images classified by the qualitative and subjective human eye (Rasp *et al*., 2019; Stevens *et al*., 2020). This subjectivity inevitably gives rise to ambiguous detections, and this is why there are a certain number of overlaps between multiple pattern rectangles. In addition to Figure 1b, we here provide two more Examples of such ambiguous classifications that were identified in previous studies (Stevens *et al*., 2020; Bony *et al*., 2020). One is

due to the confusion between the patterns Flower and Fish (Figure A1a,c) and the other one is between Gravel and Sugar (Figure A1b,d).

### A.2 Examples of known ambiguities in NN‐pattern detection

Figure A2a shows how the four patterns are distributed according to their $I_{\text{org}}/S$ values. (b, c) show the distribution functions of $I_{\text{org}}$ and S. Note that (a) is the same as figure 1 in Bony *et al*., (2020), but using a different dataset, sampling frequency and period. In particular, the shape of the $I_{\text{org}}$ and S distributions are similarly skewed toward high $I_{\text{org}}$ and low S values. However, the distribution S is here shifted toward lower values than in Bony *et al*., (2020), which is presumably due to the higher spatial resolution of GOES‐16 ABI $T_{b}$ field (2 km) than the GridSat‐B1 product (0.07°) used in Bony *et al*., (2020).

### A.3 Impact of multiple pattern overlaps on pattern frequency and cloud cover

We here test the robustness of our conclusions to the degree of overlaps between multiple NN‐detected patterns (Figures A3, A4, A5). This is done in two ways.

First, we vary a score of the neural network algorithm – which we call a *classification score* – which measures how well the detected cloud structure fits to that of a specific pattern (Figures A3 and A4). When the score is increased, the chance to detect overlapping pattern rectangles is reduced and the sum of pattern areas (including unclassified areas) approaches the domain area. The default value of the score used in this study is 0.4 (Figures A3 and A4, solid line). As shown in Figure A3, the *error* due to the overlaps is substantially reduced with a score of 0.5 (dotted line) and almost zero with a score of 0.6 (dashed line). The drawback of increasing the score is that less patterns are detected overall: in Figure A4 (right panels), the temporal frequency of pattern occurrence ($f_{k}$) is substantially reduced as the score is increased. Figure A4 (left panels) shows that the phase and amplitude of the daily cycles in cloud cover for a given pattern ($CC_{k}$) are, however, quite robust and independent of the classification score. The large differences that are seen occur essentially for a score of 0.6, for which the sample size of detected patterns per category becomes quite low (Figure A4, right panels).

Second, we compare in Figure A5 the daily cycle in pattern frequency and cloud cover between two data samples: one containing all detected patterns overlapping the BCO site at a given timestep (thus including multiple pattern overlaps), and one containing only the timesteps with single pattern detection at BCO. Figure A5 shows that the daily cycles are very simlar whether we allow multiple pattern overlaps or not.

Overall these two supplemental analyses show that multiple pattern overlaps have a minimal effect on the daily cycles of occurrence frequency and cloud cover of mesoscale patterns.

### A.4 Sensitivity of pattern cloud cover to spatial scales

Figure A6 shows the GOES‐16 ABI cloud cover for patterns overlapping BCO (as in Figure 4c), but the cloud cover averaging for each detected pattern rectangle is limited to a 20 km${\;}^{2}$ subdomain centred over BCO (Figure A6a), or located 10 km east of BCO (Figure A6b). The comparison between Figure A6 and Figure 4c shows that the daily cycles of pattern‐related cloud covers are sensitive to the spatial scale used to compute the cloud covers. Therefore, we presume similarly that the results at BCO (Figure 7) and at NTAS (Figure 8f,g) are to some extent affected by the relatively small temporal interval (used to average the surface or atmospheric field for a given pattern) compared to the size of patterns. On a somewhat different level, the comparison between Figure A6a,b shows that the results are very similar whether they are focused over BCO or east of BCO, suggesting a negligible effect from the island (e.g., land/sea breezes).
